# Supplementary material for: The M2a Macrophage Phenotype Accompanies Pulmonary Granuloma Resolution in Mmp12 Knock-Out Mice Instilled with Multiwall Carbon Nanotubes
Source: Int J Mol Sci. 2021 Oct 13;22(20):11019. doi: 10.3390/ijms222011019 (PMC8537143; doi:10.3390/ijms222011019)
Supplement: Supplementary file 1 [file ijms-22-11019-s001.zip › ijms-1395249-supplementary.pdf]

International Journal of *Molecular Sciences*

MDPI

Supplementary Materials

Figure S1:

Rosiglitazone was administered to MWCNT instilled mice as described [1]. Rosiglitazone (6mg/kg) significantly reduced the expression of MMP-12 gene expression 20 days post MWCNT instillation in C57Bl/6 mice.

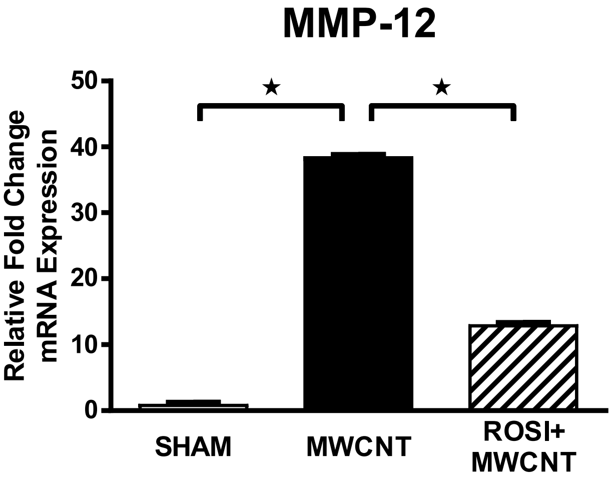

Figure S1: Rosiglitazone Reduces Alveolar Macrophage MMP–12 gene 20 days post MWCNT-Instillation in C57Bl6 (wild-type). Quantitative-PCR analysis of MMP -12 expression in BAL cells of vehicle or MWCNT-instilled animals receiving normal chow or rosiglitazone (6mg/kg). * $p \leq 0.05$: $n \geq 10$.

Reference:

1. McPeek, M.; Malur, A.; Tokarz, D. A.; Murray, G.; Barna, B. P.; Thomassen, M. J., PPAR-gamma pathways attenuate pulmonary granuloma formation in a carbon nanotube induced murine model of sarcoidosis. *Biochem. Biophys. Res. Commun* **2018,** *503* (2), 684-690.
